# Complete mitochondrial genome of a leaf beetle, *Callispa bowringi* (Coleoptera: Chrysomelidae)

**DOI:** 10.1080/23802359.2017.1413302

**Published:** 2018-02-12

**Authors:** Peng Liu, Qingyun Guo, Jiasheng Xu, Chengqing Liao, Xiaohua Dai

**Affiliations:** a Leafminer Group, School of Life and Environmental Sciences, Gannan Normal University, Ganzhou, China;; b National Navel-Orange Engineering Research Center, Ganzhou, China

**Keywords:** Leaf beetle, mitochondrial genome, *Callispa bowringi*, phylogenetic analysis

## Abstract

The complete circular mitochondrial genome of *Callispa bowringi* was 17,060 bp in length, including two ribosomal RNA genes, 22 transfer RNAs, 13 protein-coding genes (PCGs) and one 2246-bp non-coding AT-rich region. All 22 tRNA genes displayed a typical clover-leaf structure except for tRNA^Ser^ (AGN). All 13 PCGs initiated with ATN codons. Only three PCGs used the incomplete stop codons “TA” or “T”, while ten PCGs terminated with typical stop codons “TAA” and “TGA”. Phylogenetic analysis based on 13 PCGs of Chrysomelidae mitogenomes showed that *C. bowringi* was closely related to *Agonita chinensis* and *Rhadinosa nigrocyanea*.

The leaf beetle genus *Callispa* belongs to the tribe Callispini (Chrysomelidae: Cassidinae), with 173 species in Oriental and Ethiopian region (Staines 2015) and 31 species in China (Chen et al. 1986; Hua 2002). *Callispa bowringi* is widely distributed in Southern China and Southeast Asia, with many bamboo genera as its host plants (Staines 2015). The larvae and adults feed leaves on upper surface and form slender feeding channels. However, there are no studies on Callispini mitogenome. In this paper, we presented the complete mitochondrial genome of *C. bowringi* (GenBank: MG456836) based on the Illumina pair-end sequencing data. It could improve our understanding on the phylogenetic position of Callispini within the family Chrysomelidae.

The specimen was collected at Jiulianshan, Jiangxi Province, China (geographic coordinate: N 24.57, E 114.44). The adults were stored in 100% ethanol at −80 °C. Specimen and sample were deposited at Leafminer Group in Gannan Normal University. The total DNA was extracted from head tissue of the adults using Sangon animal DNA extract kit (Sangon Inc., Shanghai, China). DNA was preserved at −20 °C. De novo assemblies, contigs and scaffolds were achieved using A5-miseq v20150522 (Coil et al. 2014) and SPAdesv3.9.0 (Bankevich et al. 2012). The obtained assemblies were analyzed with mummer v3.1 (Kurtz et al. 2004) to identify syntenic regions and their contig mapping. The alignment file was corrected using pilon v1.18 (Walker et al. 2014) to get the final mitogenome sequence. Two rRNA and all protein-coding genes (PCGs) were annotated by alignment with homologous genes from other published mitochondrial sequences using Geneious R11 (Kearse et al. 2012). The tRNA genes were predicted using tRNAscan-SE (Lowe and Eddy 1997).

The circular mitogenome of *C. bowringi* was 17,060 bp, AT rich (75.62%), and included 37 genes for two ribosomal RNA, 22 tRNAs, 13 PCGs and a 2247 bp long non-coding AT-rich region. All 13 PCGs initiated with ATN codons. Only three PCGs used the incomplete stop codons “TA” or “T”, while 10 PCGs terminated with typical stop codons “TAA” and “TAG”. All 22 tRNAs had a typical clover-leaf secondary structure, except for tRNA^Ser^ (AGN) lacking a stable dihydrouridine (DHU) stem, which has been reported in several insects’ mtDNA (Kim et al. 2012; Song et al. 2017). The 16S rRNA was 1287 bp long with an AT content of 78.5%, while the 12S rRNA was 735 bp long with an AT content of 78.2%. The non-coding region with an AT content of 77.2% was known for replication initiation (Nardi et al. 2001).

The 13 PCGs was 11,023 bp in total length, with the overall AT content of 74.4%. The concatenated datasets of the 13 PCGs from mitogenome of 13 Chrysomelidae species from GenBank were adopted to build phylogenetic tree by using maximum-likelihood method. Phylogenetic analysis indicated that *C. bowringi* was grouped into the clade including *Agonita chinensis* (Guo et al. 2017b), *Rhadinosa nigrocyanea* (Guo et al. 2017a), *Laccoptera ruginosa*, and *Cassida viridis* (Figure 1), which agreed with morphological taxonomy (Chen et al. 1986; Staines 2015).

**Figure 1.**
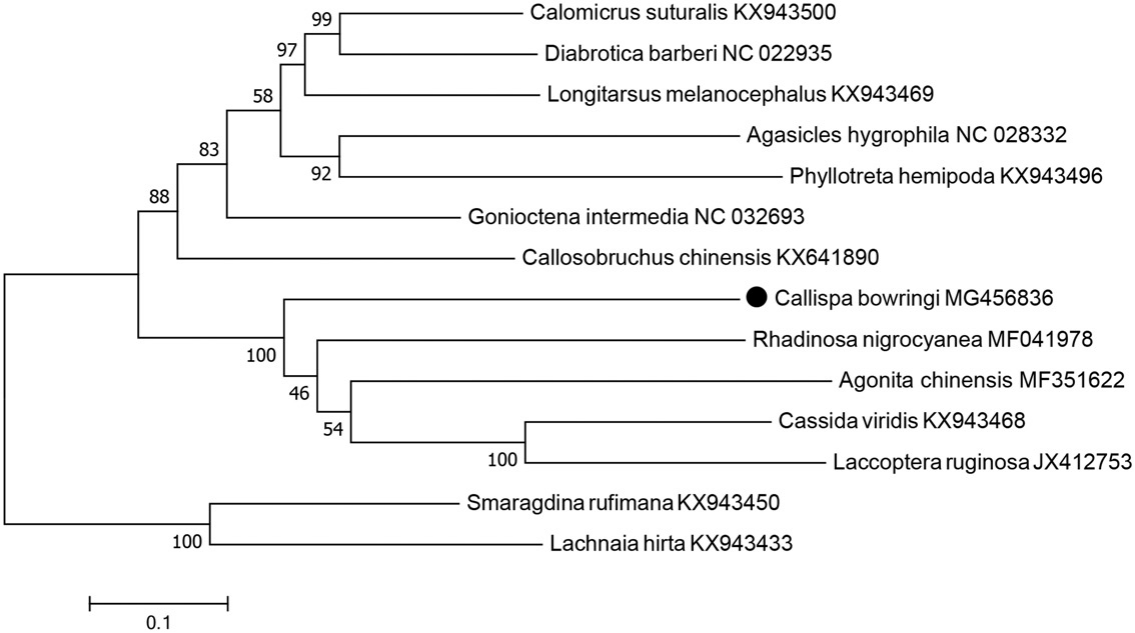
Maximum-likelihood tree of evolutionary relationships *C. bowringi* and 13 other Chrysomelidae species based on mitochondrial PCGs catenated dataset.
